# Tissue Factor/Factor XIa Dual-activated Thrombin Generation is Able to Reliably Measure Thrombin Generation at All Hemophilia A Disease Severities

**DOI:** 10.1055/a-2916-8837

**Published:** 2026-08-18

**Authors:** Tom William van de Berg, Alexandra Heinzmann, Stella Thomassen, Saskia E.M. Schols, Floor C.J.I. Heubel-Moenen, Nick van Es, Frits R. Rosendaal, Samantha Gouw, Tilman Mathias Hackeng, Erik A.M. Beckers

**Affiliations:** 1 Department of Hematology 199236 Maastricht University Medical Centre (MUMC+)MaastrichtLimburgNetherlands; 2 Department of Biochemistry 5211 Maastricht University, Cardiovascular Research Institute Maastricht (CARIM)MaastrichtLimburgNetherlands; 3 Department of Hematology 6034 Radboud University Medical CenterNijmegenGENetherlands; 4 Hemophilia Treatment Centre Nijmegen-Eindhoven-MaastrichtMaastrichtNetherlands; 5 Department of Vascular Medicine 26066 Amsterdam UMC, University of AmsterdamAmsterdamNorth-HollandNetherlands; 6 Department of Clinical Epidemiology 4501 Leiden University Medical CenterLeidenZHNetherlands; 7 Department of Pediatric Hematology 522567 Amsterdam UMC location University of AmsterdamAmsterdamNoord-HollandNetherlands

**Keywords:** hemophilia A/B, thrombin, factor VIII

## Abstract

**Introduction** Predicting bleeding in hemophilia A is difficult due to limitations of existing factor VIII (FVIII) activity assays, which correlate poorly with clinical bleeding tendency. Measurement of thrombin generation (TG) by calibrated automated thrombography (CT) may be an alternative to predict bleeding as it measures an individual’s complete plasma derived coagulation potential, rather than just focusing on one part of the coagulation cascade. However, lack of standardization limits its applicability.

**Objective** To evaluate the ability of our standardized tissue factor (TF)/factor XI (FXI) a dual-activated thrombin generation assay (TGA) to assess TG across severe, moderate, and mild hemophilia A. Secondarily, we evaluate whether varying TG levels at similar FVIII concentrations translate to different clinical bleeding tendencies.

**Materials and Methods** Plasma samples from 657 adult patients with hemophilia A of all severities included in the Hemophilia in the Netherlands 6 (HiN6) study were used. Samples were collected through standardized protocols from six Dutch hemophilia treatment centers, and stored in a centralized biobank. FVIII activity was centrally measured using one-stage assays. Thrombin generation was measured using our in-house TF/FXIa TGA protocol using 1 pm TF, 100 pm FXIa, and 30 μM phospholipids. Bleeding tendencies were analyzed based on patient questionnaires.

**Results** The TF/FXIa dual-activated TGA protocol was able to measure thrombin generation across all severities of hemophilia A. Median peak heights (PH) were 119.5, 98.9, and 214.7 nM for severe, moderate, and mild, respectively. Moreover, the assay showed substantial variation in interindividual TG levels among patients with comparable FVIII activity levels (coefficient of variance of 99%, 83%, and 47% for severe, moderate, and mild hemophilia A, respectively). However, TG did not differentiate between patients with and without self-reported bleeding in this study.

**Conclusion** The standardized TF/FXIa dual-activated TGA represents a tool for assessing individual coagulation potential in hemophilia A. More research is needed to further characterize the interindividual differences in thrombin generations at similar FVIII levels and possibly link these results to bleeding tendency.

## Introduction

Measurement of the coagulation capacity in patients with hemophilia A is difficult. Coagulation factor VIII (FVIII) activity assays, in contrast to the thrombin generation assay (TGA), are unable to assess complex interplay of the different coagulation proteins in the coagulation cascade. Thrombin generation has been used for approximation of FVIII activity, but it correlates poorly with clinical bleeding outcomes, especially in the range of FVIII:C below 5%. 1 So far both thrombin generation and FVIII assays have been unsuccessful in explaining the differences in bleeding tendency at similar FVIII levels, which is still one of the major challenges in hemophilia care. 2

Global coagulation assays such as the measurement of thrombin generation by calibrated automated thrombography (CT) may better reflect the innate coagulation potential of specific individual patients. 3 4 5 6 Although thrombin generation is increasingly used in the analysis of patients with hemophilia A, standardization of (pre-)analytical variables is often lacking. These (pre-)analytical variables, such as contact activation, phospholipid mixture, and the type and concentration of different coagulation triggers, can profoundly influence the outcomes of the assay. 7 This influence may even be greater in the setting of severe hemophilia A (<1% FVIII) due to the low amount of innate coagulation potential of the patient compared with moderate (1–5% FVIII) or mild (5–40% FVIII) hemophilia A patients. In addition, tissue factor activated thrombin generation in severe hemophilia A samples is largely driven by the extrinsic tenase complex. Combining extrinsic (TF) with intrinsic (FXIa) activation of thrombin generation might overcome this challenge. As such, we previously published a TF/FXIa dual-activated protocol for thrombin generation in severe hemophilia A patients. 8 Although similar approaches have been published since, they are often used in smaller study population. 9 In this paper, we report the results of our TF/FXIa dual-activated thrombin generation protocol in the Hemophilia in the Netherlands 6 (HiN6) study, a large multicenter cohort of patients with hemophilia A. We assessed whether the assay is able to measure thrombin generation in hemophilia A plasma samples of all disease severities. Additionally, we assessed whether the assay is able to differentiate thrombin generation levels between hemophilia A patients at similar FVIII levels. Lastly, we investigate whether our TGA can distinguish patients based on bleeding phenotype severe hemophilia A.

## Materials and Methods

### HiN6

The validation study of the TF/FXIa dual-activated TGA is an independent substudy of the multicenter cross-sectional HiN6 study, a nation-wide questionnaire-based study on hemophilia patients. In addition, HiN6 stored plasma samples for sub-studies in a centralized biobank. Plasma samples used within this substudy were provided by the HiN6 biobank. The study was approved by the Medical Ethical Committee (NL59114.058.17) Leiden University Medical Centre (LUMC). All participants provided written informed consent. As part of the study patients were required to fill out a questionnaire detailing disease-related events (among others: bleeding, hospitalization, medication use) and management of the disease in the past 12 months.

### Patients

We included 657 patients with hemophilia A from the HiN6 study into our substudy. Inclusion criteria for our substudy were patients ≥18 years of age, hemophilia A (all severities), one-stage FVIII activity measurement in the study sample, and the availability of a plasma sample. Exclusion criteria were pediatric patients, hemophilia B, and inadequate amount of plasma provided. In the subsequent analysis on bleeding tendency, patients who did not complete the bleeding tendency subsection of the HiN6 questionnaire were excluded. Inclusions for this study were done before the introduction of emicizumab in the Netherlands.

### Blood Sampling

Blood sampling was performed in each participating hemophilia treatment center according to predefined standardized protocols. Samples were centrifuged twice for 15 min at 3000 g at room temperature after which the plasma was stored at −80 °C. If clinically possible, a FVIII washout period was requested. Blood samples were aimed to be taken at least 72 h after the last administration of FVIII replacement therapy or desmopressin (DDAVP), and at least 7 days after the resolution of a bleeding episode. Samples were stored in a centralized biobank. One-stage FVIII activity measurements were done centrally by one-stage FVIII measurement for all study samples according to the HiN6 study protocol.

### Thrombin Generation Assay

The TF/FXIa dual-activated thrombin generation was performed as previously published. 8 In short, thrombin generation was measured by CT (Thrombinoscope BV, Maastricht, The Netherlands) 10 in the presence of 1 pm of tissue factor (TF), 100 pm FXIa (Innovatiove Research, Michigan, USA), and 30 μM phospholipids (PL) 20%/60%/20% phosphatidylserine/phosphatidylcholine/phosphatidylethanolamine at a temperature of 37 °C. Thrombin generation was assessed by addition of Z-Gly-Gly-Arg 7-amino-4-methylcoumarin low affinity fluorescent substrate. The TF/PL/FXIa mixture was incubated for 1 h. Each measurement well consisted of 80 μL patient plasma, 20 μL TF/PL/FXIa mix, and 20 μL of CaCl _2_ and fluorescent substrate. Measurements were performed using the fluoroskan ascent reader (Thermo Labsystems, Helsinki, Finland) utilizing a 390/460 nM filter and were consecutively calculated by Thrombinoscope software. Each sample was measured as duplicate. Means were presented with standardized deviation. We compared patients with severe (FVIII < 1%) and moderate (FVIII 1–5%) hemophilia to those with mild hemophilia (FVIII 5–40%).

### Data Analysis

Data analysis and creation of all figures were performed with Graphpad Prism v10.0.1 (Graphad Software, LLC) and IBM SPSS Statistics 28 (IBM Corp. Released 2021. IBM SPSS Statistics for Windows, Version 28.0. Armonk, NY: IBM Corp). The primary outcome measure was peak height (PH). PH was shown relative to measured FVIII levels. Means were presented with SD. We compared patients with severe (<1% FVIII) and moderate (1–5% FVIII) hemophilia to those with mild (1–40% FVIII) hemophilia (based on lowest historical FVIII value). We assessed the peak thrombin height according to the measured FVIII activity level in the sample, and categorized according to hemophilia severity and bleeding history (those who had at least one bleed of any type that required additional therapy in the past 12 months). Confidence intervals are CI95%. We also performed a sub-analysis of severe hemophilia A patients on prophylaxis with reported bleeds in the last 12 months versus patients in this same group with no reported bleeds, trying to differentiate bleeders versus non-bleeders based solely on TGA results. PH was chosen as primary outcome. Endogenous thrombin potential (ETP; area under the curve) showed similar results to PH as shown in **Supplementary Fig. S1** (online only).

## Results

### Patient Characteristics

Samples from 657 patients with hemophilia A from the HiN6 study were used. Patient characteristics are listed in Table 1 . Most patients (86%) with severe hemophilia as well as some patients with moderate and mild hemophilia A reported the use of prophylaxis. Annualized bleeding rates (ABR) were higher in patients with severe disease (3.04/year) compared with moderate or mild hemophilia A patients (2.56/year and 0.52/year respectively).

**Table 1:** Patient characteristics.

	Hemophilia A ( *n* = 657)	Mild ( *N* = 294)	Moderate ( *N* = 97)	Severe ( *N* = 266)
Patient characteristics				
Mean age in years (SD)	48.62 ± 18.30	52.79 ± 17.66	47.76 ± 18.30	44.54 ± 18.01
Length in cm (SD)	181.39 ± 8.4	182.04 ± 7.6	181.59 ± 7.2	180.62 ± 9.5
Weight in kg (SD)	84.67 ± 14.8	85.99 ± 14.3	84.30 ± 12.7	83.37 ± 16.1
Patients using prophylaxis (%)				
Yes	166 (25%)	5 (2%)	12 (12%)	149 (56%)
No	261 (40%)	179 (61%)	58 (60%)	24 (9%)
Unknown	230 (35%)	110 (37%)	27 (28%)	93 (35%)
Inhibitor (%)				
Never	317 (48%)	123 (42%)	55 (57%)	139 (52%)
Past	36 (5%)	11 (4%)	3 (3%)	22 (8%)
Current	19 (3%)	10 (3%)	4 (4%)	5 (2%)
Unknown	285 (43%)	150 (51%)	35 (36%)	100 (38%)
Blood group (%)				
O	240 (37%)	98 (34%)	48 (49%)	94 (35%)
A	194 (30%)	69 (23%)	24 (25%)	101 (38%)
B	47 (7%)	21 (7%)	4 (4%)	22 (8%)
AB	19 (3%)	9 (3%)	2 (2%)	8 (3%)
Unknown	157 (24%)	97 (33%)	19 (20%)	41 (16%)
Joint bleeding ^a^ (%)				
Yes	152 (23%)	17 (6%)	26 (27%)	109 (41%)
No	418 (64%)	232 (79%)	63 (65%)	123 (46%)
Unknown	87 (13%)	45 (15%)	8 (8%)	34 (13%)
Mucosal bleeding ^a^ (%)				
Yes	21 (3%)	10 (3%)	3 (3%)	8 (3%)
No	549 (84%)	239 (82%)	86 (89%)	224 (84%)
Unknown	87 (13%)	45 (15%)	8 (8%)	34 (13%)
Muscle bleeding ^a^ (%)				
Yes	93 (14%)	26 (9%)	17 (18%)	50 (19%)
No	477 (73%)	223 (76%)	72 (74%)	182 (68%)
Unknown	87 (13%)	45 (15%)	8 (8%)	34 (13%)
Wound bleeding ^a^ (%)				
Yes	61 (9%)	25 (9%)	15 (15%)	21 (8%)
No	509 (78%)	224 (76%)	74 (76%)	211 (79%)
Unknown	87 (13%)	45 (15%)	8 (8%)	34 (13%)
Other bleeding ^a^ (%)				
Yes	29 (4%)	14 (5%)	5 (5%)	15 (6%)
No	536 (82%)	235 (80%)	84 (87%)	217 (81%)
Unknown	87 (14%)	45 (15%)	8 (8%)	34 (13%)

Note: ^a^ Bleeding reported as bleeds in the last 12 months.

### Thrombin Generation Assay (TGA)

**Fig. 1:**
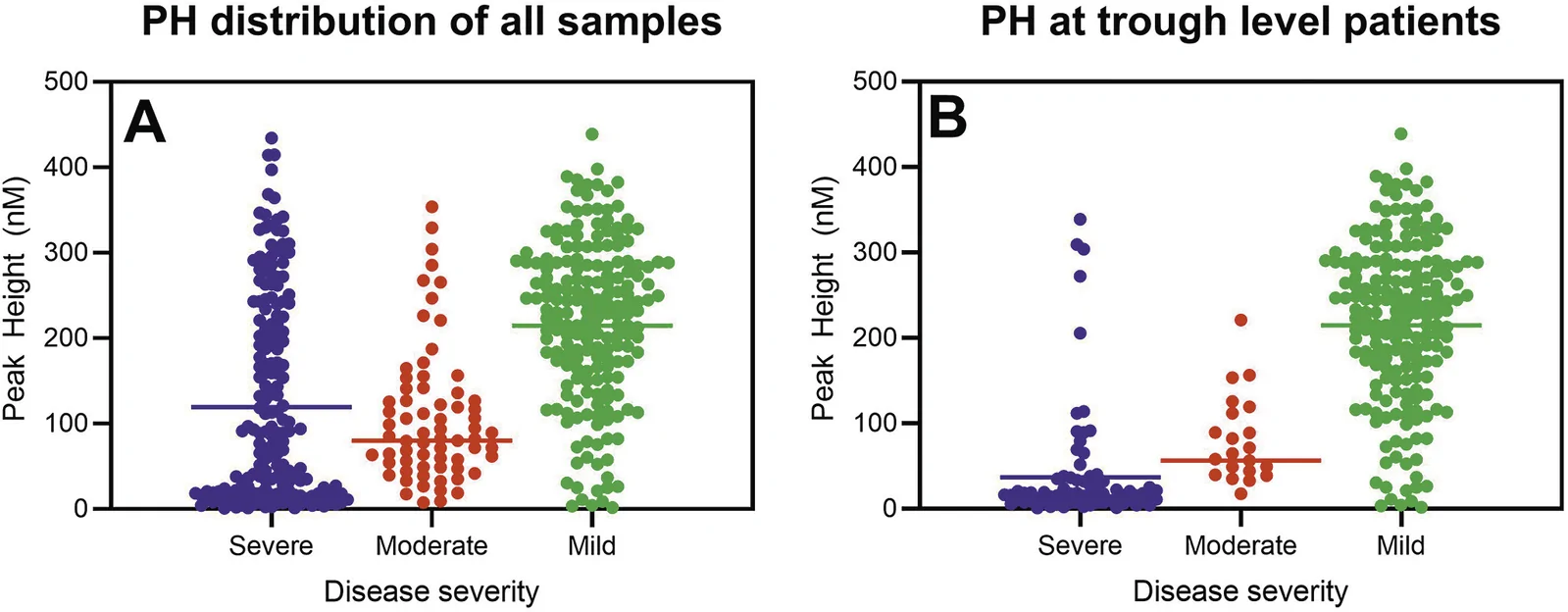
Distribution of all measured peak heights in hemophilia A plasma categorized by disease severity ( **A** ), a sub-selection of patients at trough levels of FVIII is shown in ( **B** ). Severe (blue), moderate (red), and mild (green) hemophilia A. PH, peak height.

The distribution of PH within the study group stratified by disease severity is shown in Fig. 1A . Mean PH was 119.5 nM (CI 102.7, 136.2) for severe, 98.9 nM (CI 79.95, 117.8) for moderate, and 214.7 nM (CI 200.6, 228.8) for mild hemophilia A patients. The wide variation in PH observed in the severe and moderate hemophilia A group is explained by the fact that trough levels were obtained in only a minority of samples (31.5% in severe hemophilia A; 24.7% in moderate hemophilia A). Fig. 1B shows a subset of those patients at trough levels of FVIII replacement therapy at the time of blood withdrawal. Mean PH was 36.8 nM (CI 22.0, 52.6) for severe, 68.1 nM (CI 45.1, 51.6) for moderate, and 214.7 nM (CI 200.6, 228.8) for mild hemophilia A patients. Interestingly, substantial variation in thrombin generation within each disease severity group (severe, moderate, and mild hemophilia A), independent of FVIII levels, is seen between patients. ETP showed a similar pattern and is included as **Supplemental Fig. S1** (online only).

**Fig. 2:**
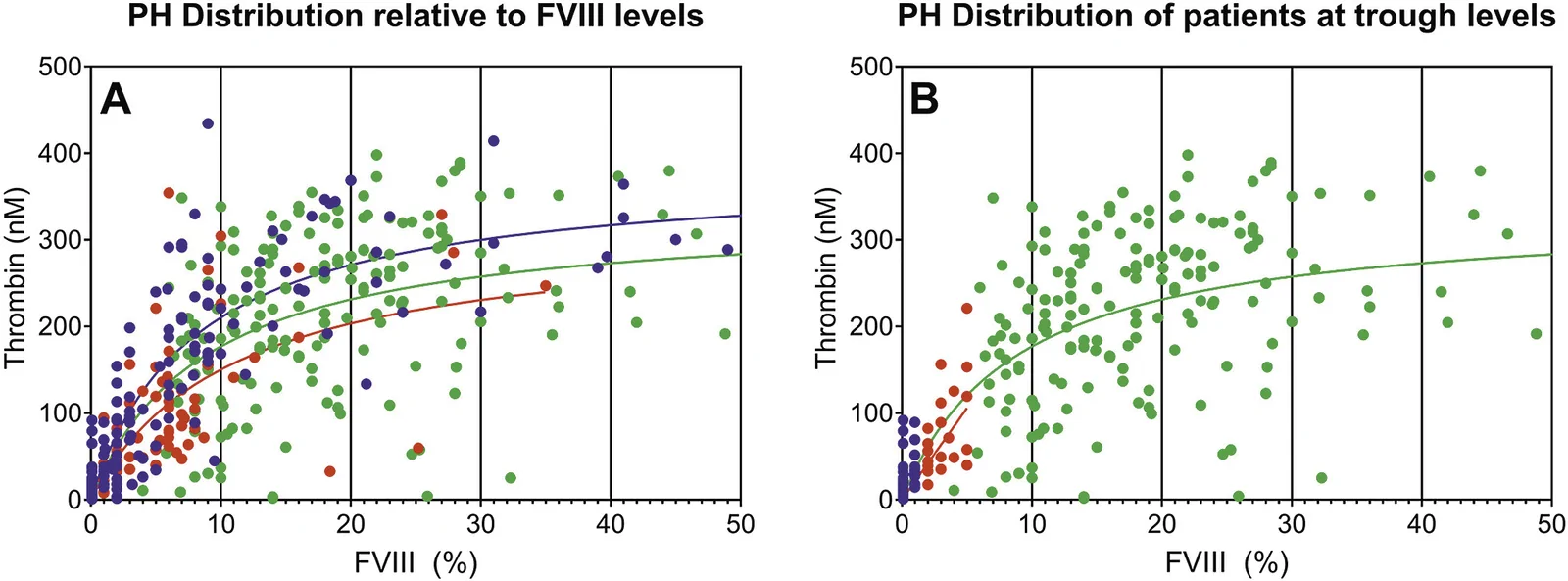
Peak height distribution with increasing FVIII levels in all severe (blue), moderate (red), and mild (green) hemophilia A patients ( **A** ) and patients at trough levels of FVIII-replacement therapy only ( **B** ). F, factor; PH, peak height.

**Fig. 3:**
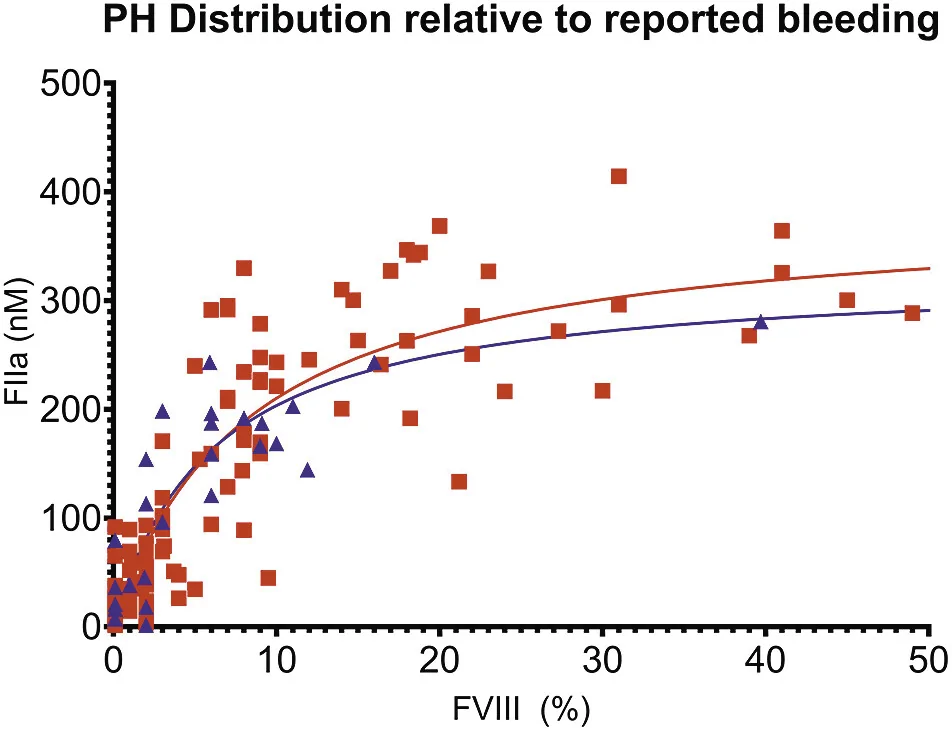
Distribution of peak height in hemophilia A (HA) patients with at least one reported bleed in the last 12 months (red) versus non-bleeders (blue) relative to their FVIII levels at the time of measurement.

A non-linear fit model was applied to visualize the trend of thrombin increase of thrombin generation relative to FVIII concentration ( Fig. 2A ). The increase in thrombin generation was most prominent at FVIII levels in the lower ranges (FVIII < 20%), after which the PH plateaued at FVIII activity of 30–40%. This trend remained consistent when only taking into account patients at trough levels of FVIII ( Fig. 2B ). In a subgroup of 183 patients with severe hemophilia on prophylactic therapy who filled in the bleeding questionnaire, we found no difference in thrombin peak between severe hemophilia A patients with (mean PH 120.7 nM; CI 100.8, 140.6 nM) and those without any bleeds in the last 12 months (mean PH 119.5 bN; CI 84.91, 154.2 nM) ( Fig. 3 ). Bleeders were defined by patient questionnaires as having at least one bleed of any type that required additional therapy in the past 12 months. Even with use of more stringent criteria to define bleeders (e.g., >3 bleeds per year), no significant difference was seen (data not shown). Fig. 3 shows the PH of bleeders versus non-bleeders regarding their FVIII levels further visualized with a non-linear fit for both groups. There was no significant difference between the groups.

## Discussion

In this post-hoc analysis of the Hemophilia in Netherlands study, we applied the previously established TF/FXIa dual-activated TGA protocol in a large group of hemophilia A patients, aiming to validate this thrombin generation protocol. 8 With this TF/FXIa dual-activated TGA protocol within the HiN6 study population, we were able to measure thrombin generation in hemophilia A patients of all disease severities. In line with our in vitro data the TGA protocol showed good sensitivity to FVIII changes, especially at clinically relevant FVIII levels below 20%. 2

In this HiN6 substudy cohort substantial variation in thrombin generation was evident, even within disease severity groups (severe, moderate, mild) as seen in Fig. 1 . The performed one-stage FVIII testing showed a wide variety of FVIII levels within even the severe population, as patients were sometimes not able or not willing to accept a washout period ( Fig. 1A ). However, even at the lowest measured FVIII levels by one-stage testing ( Fig. 1B ), differences in PH remained. Interestingly, also within the mean PH of the moderate or mild groups a wide variation in PH was observed, further strengthening the question whether these differences are due to alternative proteins contributing to coagulation in these patients or due to pre-analytical variability. Indeed, these variations might exist due to several (pre-)analytical variances within patients, the study protocol, or perhaps within non-FVIII coagulation proteins. It is important to recognize the pre-analytical variables within the study. Blood samples were drawn and handled in each participating hospital based on their local protocols, possibly leading to variations in contact activation or protein degradation due to handling. Additionally, comparison between patients of the same disease severity is hampered by the great variation in FVIII levels due to incomplete washout in a substantial portion of the study population. The latter also brings up the discussion whether all FVIII replacement therapies behave similarly within the current thrombin generation setup, possibly adding yet another layer of variability. Perhaps, these differences are partially due to non-FVIII related plasma proteins like other clotting factor deficiencies or differences in anticoagulant proteins such as protein C or tissue factor pathway inhibitor (TFPI). It is possible that this variability in pre-analytical variables might mask subtle biological differences between patients. However, based on the current study, no such conclusion could be made; more research on this is needed. This data further characterizes individual thrombin generation potential corrected for FVIII and disease severity compared with a previous study by Verhagen et al. using the same HiN6 cohort. 11 In line with our published in vitro data, the relative increase in thrombin generation was highest at lower FVIII levels (<20%), then forming a plateau at around 30 to 40% FVIII. 8 This is in contrast to TF-activated thrombin generation in hemophilia A. 8 The increased sensitivity could possibly lead to better characterization of coagulation in hemophilia A patients at low FVIII levels. Such characterization of coagulation within hemophilia A patients with TF/FXIa might provide more information compared with TF-only protocols. A limitation of the current setup is the lack of data on healthy individuals, as a comparison mainly to patients at high FVIII levels. However, as our aim was to validate the thrombin generation protocol, control samples within our measurements consisted of pooled severe hemophilia A samples rather than healthy controls. It would be interesting for further research to compare healthy individuals with hemophilia A patients at high FVIII levels to see whether coagulation fully normalizes or that a possible difference in coagulation potential could be better defined.

The secondary aim of the paper was to investigate whether the TF/FXIa dual-activated TGA would allow for measurement of discrete differences in thrombin generation between patients at low FVIII levels, allowing for prediction of bleeding phenotype compared with already promising previous studies. 3 4 5 12 However, we were unable to show differences in thrombin generation between patients with versus without bleeding in the prior 12 months. Although our analysis was not able to connect lower thrombin generation to a bleeding phenotype, it is subject to major limitations. There was a large heterogeneity of prophylactic regimens within the study; data on long-term bleeding history or joint damage was unavailable and the majority of the data included only the amount of bleeds in the past 12 months. This, once again, emphasized the difficulty of truly determining bleeding phenotype, as it remains such a multivariable expression of severe bleeding disorders. Based on the current data, taking into account the (pre-)analytical variables as presented in this paper, no definite conclusion can be drawn regarding thrombin generation and clinical bleeding phenotype. More research is needed to further explore whether differences in bleeding tendency can be measured using the TF/FXIa dual-activated TGA. Ideally these studies should take into account more confounders of bleeding tendency such as lifetime bleeds and differentiate more between traumatic and non-traumatic bleeds and the type of prophylaxis. Additionally, more available coagulation-related biomarkers such as VWF levels, coagulation factor levels, alternate clotting test, and inflammatory markers might offer more insight into the state of coagulation within these patients. Future research should focus on trying to provide a clear picture of the biochemical and clinical state of hemophilia patients to more precisely define their bleeding tendency.

## Conclusion

The optimized, dual-activated TF/FXIa TGA protocol for the measurement of hemophilia A patients was investigated in a large cohort. The assay was able to measure thrombin generation at all FVIII levels. The assay showed feasibility of applying the TF/FXIa dual-activated TGA in large clinical cohorts. The assay was able to visualize interindividual differences in thrombin generation at the same FVIII levels in all disease severities, which need to be further characterized. Future research should focus on further characterizing the determinants of these interindividual differences in thrombin generation at similar FVIII levels and if these differences correlate with differences in bleeding tendency. The dual-activated TF/FXIa TGA protocol is able to characterize coagulation in hemophilia A patients; however, more research is needed to define the interindividual differences in measured thrombin generation at similar FVIII levels.

## Supplementary Material

Ergänzendes Material Supplementary Material
