# Supplementary material for: Tissue Factor/Factor XIa Dual-activated Thrombin Generation is Able to Reliably Measure Thrombin Generation at All Hemophilia A Disease Severities
Source: TH Open. 2026 Aug 18;10:a29168837. doi: 10.1055/a-2916-8837 (PMC13492305; doi:10.1055/a-2916-8837)

Supplementary Fig. S1

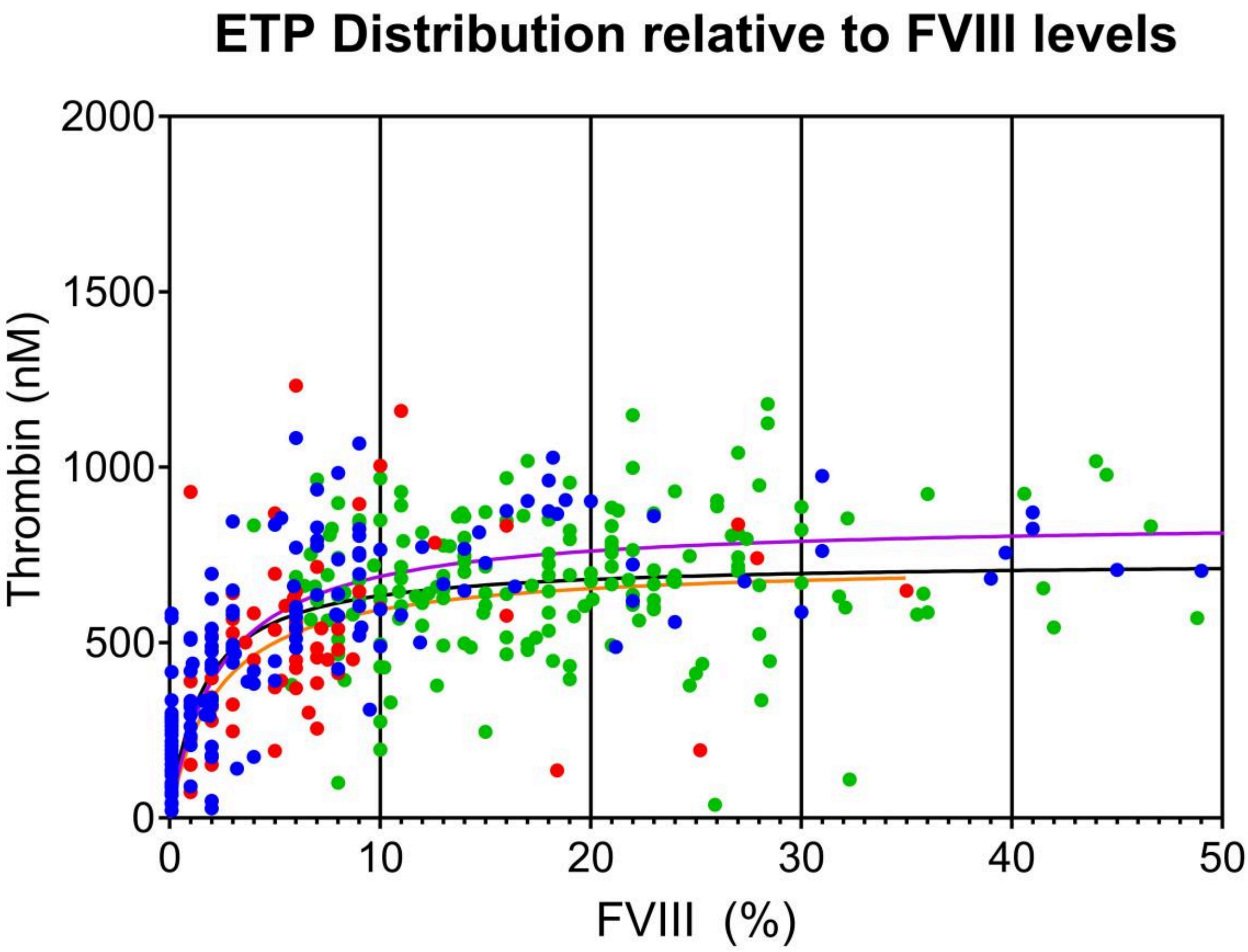

Supplement: Supplementary file 1 [file 10-1055-a-2916-8837_29389397.pdf]
